# Dietary behaviors, physical activity and sedentary lifestyle associated with overweight and obesity, and their socio-demographic correlates, among Pakistani primary school children

**DOI:** 10.1186/1479-5868-8-130

**Published:** 2011-11-25

**Authors:** Muhammad Umair Mushtaq, Sibgha Gull, Komal Mushtaq, Ubeera Shahid, Mushtaq Ahmad Shad, Javed Akram

**Affiliations:** 1 Ubeera Memorial Research Society, Allama Iqbal Medical College, Lahore, 54000 Punjab, Pakistan; 2 District Health Office Nankana Sahib, Punjab Department of Health, Nankana Sahib, 39100 Punjab, Pakistan

## Abstract

**Background:**

There is no data on diet- and activity-related behaviors associated with overweight and obesity among Pakistani school-aged children. The study aimed to explore dietary behaviors, physical activity and sedentary lifestyle associated with overweight and obesity, and their socio-demographic correlates, among Pakistani primary school children.

**Methods:**

A population-based cross-sectional study was conducted with a representative multistage random cluster sample of 1860 children aged five to twelve years in Lahore, Pakistan. Overweight (> +1 SD) and obesity (> +2 SD) were defined using the World Health Organization reference 2007. Chi-square test was used as the test of trend. Linear regression was used to examine the predictive power of independent variables in relation to body mass index (BMI). Logistic regression was used to quantify the independent predictors and adjusted odds ratios (aOR) with 95% confidence intervals (CI) were obtained. Statistical significance was considered at P < 0.05.

**Results:**

Children skipping breakfast (8%), eating fast food and snacks ≥ once a week (43%) and being involved in sedentary lifestyle > one hour a day (49%) were significantly more likely to be overweight and obese while those participating in physical activity > twice a week (53%) were significantly less likely to be overweight and obese (all P < 0.01). Skipping breakfast (P < 0.001), eating fast food and snacks (P = 0.001) and sedentary lifestyle (P < 0.001) showed an independent positive association with BMI while physical activity showed an independent inverse association (P = 0.001). Skipping breakfast (aOR 1.82, 95% CI 1.22-2.71), eating fast food and snacks ≥ once a week (OR 1.41, 95% CI 1.07-1.86), physical activity > twice a week (aOR 0.49, 95% CI 0.34-0.70) and sedentary lifestyle > one hour a day (aOR 1.56, 95% CI 1.19-2.03) were independent predictors of being overweight. Skipping breakfast had independent inverse association with physical activity (aOR 0.63, 95% CI 0.45-0.89) and eating fast food and snacks had independent positive association with sedentary lifestyle (aOR 1.79, 95% CI 1.49-2.16). Female gender was independently associated with skipping breakfast (aOR 1.50, 95% CI 1.04-2.16). Male gender (aOR 1.64, 95% CI 1.33-2.02), urban area with high SES (aOR 5.09, 95% CI 3.02-8.60) and higher parental education (aOR 1.74, 95% CI 1.12-2.68) were significant independent predictors of eating fast food and snacks ≥ once a week. Living in the rural area was independently associated (aOR 2.51, 95% CI 1.71-3.68) with physical activity > twice a week. Male gender (aOR 1.60, 95% CI 1.31-1.95), urban area with low SES (aOR 1.46, 95% CI 1.02-2.09), high-income neighborhoods (aOR 1.52, 95% CI 1.02-2.25), higher parental education (aOR 1.55, 95% CI 1.03-2.34) and fewer siblings (aOR 1.38, 95% CI 1.10-1.73) were independent predictors of sedentary lifestyle > one hour a day.

**Conclusions:**

Dietary behaviors, physical activity and sedentary lifestyle are independent predictors of overweight and higher BMI among Pakistani primary school children, and are significantly affected by the child's socio-demographic characteristics. These findings support the urgent need to develop a National strategy for diet and physical activity and to implement culturally relevant behavioral interventions in the resource-poor developing country settings.

## Background

Obesity is a global epidemic and children are the worst affected with an estimated ten percent of school-aged children being overweight and one quarter of these being obese worldwide [1,2]. The 2004 World Health Assembly at Geneva called for specific actions to halt the epidemic that is now penetrating the developing countries including Pakistan, mainly in the affluent urban population [3,4]. Targeted interventions, tailored to local circumstances and involving communities, should begin early in life [5]. Association of overweight and obesity with diet- and activity-related factors is important in terms of implementing effective interventions. Dietary and lifestyle behaviors are modifiable and have therefore been targets of obesity research and prevention [6,7]. Association of dietary behaviors, physical activity and sedentary lifestyle with childhood obesity has been extensively explored among school-aged children globally; however, most studies were conducted in the developed countries and literature in this regard is scarce in South Asian children. There is no data on diet- and activity-related behaviors associated with overweight and obesity among Pakistani school-aged children. Only one study conducted in Karachi, Pakistan reported physical activity levels in school-aged children and its inverse association with overweight and obesity [8]. The study aimed to explore dietary behaviors, physical activity and sedentary lifestyle associated with overweight and obesity, and their socio-demographic correlates, among Pakistani primary school children.

## Methods

### Design, setting and sample

A population-based cross-sectional study titled the Nutritional Assessment among School-going Children in Lahore, Pakistan (NASCL) was conducted among primary school children aged five to twelve years in 2009-2010. Lahore, a metropolis with multiethnic populations, is the capital of Pakistan's most populous province Punjab. It has a population of nine million including 2.5 million primary school children, and 81% of the population resides in the urban area (Administrative data, Government of the Punjab, 2010).

A stratified multistage random cluster sample of 1860 children aged five to twelve years in twelve primary schools of City District Lahore was enrolled. Stratified sampling, based on the population and educational system characteristics, was used to have proportionate representation of gender, area of residence and socioeconomic status (SES). The list of all the public and private primary schools in Lahore was provided by the Punjab Department of Education. The listed schools were stratified according to the geographic area and monthly fee structure of schools into the following four strata: a) urban with high SES (urban area and fee > 2500 PKR), b) urban with middle SES (urban area and fee = 1000-2500 PKR), c) urban with low SES (urban area and fee < 1000 PKR), and d) rural with low/disadvantaged SES (rural area and fee ~100 PKR or free). The former two strata included private (including public-private mix) schools and the later two strata included public schools. In Pakistan, public schools cater low SES urban and rural children while high and middle SES urban children are educated in private and public-private mix schools. Three schools were selected at random from each stratum and contacted by the Departments of Education and Health to participate voluntarily in the study. If the school administration refused to participate, the next school was selected randomly from the respective stratum. For each school, a list of all classes in five grades (one to five) was obtained and one class in each grade was selected at random. In this way, sixty classes, five from each school, were selected. For each of the selected classes, first thirty-one children on class attendance register, present on data collection day and aged five to twelve years, were included in the study. Children suffering from any known metabolic syndrome (e.g. Prader-Willi syndrome) and those not willing to participate in the study were excluded.

Sample size was calculated using Epi Info 6.04d (United States' Centers for Disease Control and Prevention, 2004) with a confidence (1-α) of 95%, anticipated prevalence of 5% and margin of error of ± 1. The minimum sample size calculated was 1823 and a sample of 1860 was deemed sufficient.

### Data Collection

Sampled schools were visited on pre-arranged dates in summer 2009 by a team of trained senior medical students lead by the Principal Investigator. Health education of children and teachers was also carried out after data collection in the respective school. Analogue physician health scales, standardized before the examination, were used [9]. Height in centimeters (cm) and weight in kilograms (kg) were measured following the standard procedure to the nearest 0.1 cm and 0.5 kg respectively. All measurements were taken in light summer school uniform without shoes during mornings or early afternoons. Most frequently used measure for obesity is body mass index (BMI), defined as weight (kg) divided by height squared (m^2^), and BMI-for-age is the anthropometric index of relative weight recommended by the international expert committees [10].

Study instrument was a structured questionnaire designed in English that included the following sections: a) demographic information (gender, date of birth, residential address and parental education), b) family-based characteristics (parental working status, number of siblings and number of persons in child's living room), c) dietary behaviors (breakfast, lunch, family supper and fast food and snacks), d) physical activity, and e) sedentary lifestyle. Study instruments and procedures were pre-tested in the field and modified accordingly. First section of the questionnaire that included demographic information was completed by obtaining data for all officially enrolled students in the sampled classes from school record and class teachers. Other sections of the questionnaire including family-based characteristics, dietary behaviors, physical activity and sedentary lifestyle were completed by interviewing the sampled children. Children were interviewed in presence of their class teacher (guardian) by senior medical students trained in the interviewing techniques, and the responses were based on self-recall.

Parental education level was based on the parent with the highest total years of schooling and neighborhood income level was based on the average income estimate of child's residential area obtained from the Revenue Department of City District Government Lahore [11]. Each child was asked regarding whether his/her mother works outside or is she a housewife? how many older/younger siblings he/she has? and how many persons are living in his/her living room? Information regarding breakfast, lunch, family supper and fast food and snacks a child had in the past week was collected. Although intra-individual variability precludes the use of a single recall as an accurate representation of individual dietary intake, recalls provide a valid assessment of group-level mean intake among school-aged children [12,13]. Physical activity included running or jogging, cycling, housework or yard work, and sports involving physical movement e.g. jumping rope, climbing stairs, roller-skating, swimming, cricket, soccer, badminton, basketball, handball, etc., and the frequency of participation in physical activity was measured per week [14]. Proxy measures had been shown to be predictive of physical activity in children and the most common method for measuring physical activity is a self-report survey [15,16]. Children were asked about the time in the past week they had spent in organized and other moderate- to vigorous-intensity physical activity of at least sixty minutes outside the school (this total duration does not have to be consecutive and briefer bouts can be added up). Sedentary lifestyle included television viewing, working on computer and playing video games, and it was measured as the number of hours spent daily outside the school [17]. Children were asked about television viewing and computer or video game use during the past week, and those who provided a positive answer were asked about the frequency of event during one of those days.

Informed consent statement was printed on the study forms. Verbal informed consent for the child to participate in the study was taken from class teachers and school heads. As the study involved no invasive procedure, verbal informed consent was deemed sufficient. The study was approved by the Ethical Review Board of Allama Iqbal Medical College, Lahore. Permissions to conduct the study were granted by the Punjab Departments of Education and Health, and the sampled schools.

### Statistical analysis

Data were entered and analyzed by manual and computerized checking using SPSS version 18.0 (SPSS Inc. Chicago IL, United States, 2009). Age was calculated to the precise day by subtracting the date of birth from the date of examination. The z-score values for BMI-for-age were calculated by using the World Health Organization's software, AnthroPlus, for assessing growth of the world's children and adolescents (WHO, 2009). Overweight (> +1 SD) and obesity (> +2 SD) were defined using the WHO child growth reference 2007 [18,19].

Bivariate analysis, using chi-square test as the test of trend, was conducted to compare the differences in prevalence of overweight and obesity with respect to dietary behaviors, physical activity and sedentary lifestyle. Linear regression analysis, controlled for age and gender, was used to explore the predictive power of dietary behaviors, physical activity and sedentary lifestyle significantly associated with overweight (independent variables) in relation to BMI (dependent variable). Crude odds ratios (OR) with 95% confidence interval (CI) were calculated to examine the relationship between overweight (dependent variable) and dietary behaviors, physical activity and sedentary lifestyle (independent variables) by univariate analyses. Multivariate logistic regression analysis, controlled for age and gender, was used to estimate the simultaneous effect of several covariates on a dichotomous outcome. Independent variables were entered into the multivariate model concurrently to quantify the independent predictors of overweight and adjusted odds ratios (aOR) with 95% CI were obtained. Skipping breakfast, eating fast food and snacks once or more a week, physical activity more than twice a week and sedentary lifestyle more than one hour a day were independently associated with overweight and higher BMI and socio-demographic correlates of these factors were investigated by bivariate analysis using chi-square test. Crude odds ratios (OR) with 95% confidence interval (CI) were calculated by univariate analyses. Multivariate logistic regression analyses, simultaneously adjusted for socio-demographic factors significantly associated with skipping breakfast, eating fast food and snacks once or more a week, physical activity more than twice a week and sedentary lifestyle more than one hour a day, were conducted to quantify the independent predictors and adjusted odds ratios (aOR) with 95% CI were obtained. Statistical significance was considered at P < 0.05 and all tests were two-sided.

## Results

The study included a sample of 1860 primary school children aged five to twelve years that included 20% children from each grade (one-five) and 25% children from each area and SES stratum (urban with high, middle and low SES and rural with low/disadvantaged SES). Male-female ratio was 1.11 with 52.5% boys and 47.5% girls. Median age (range) was 8 (5-12) years and mean age (SD) was 8.49 (1.81) years. Mean (SD) BMI was 20.7 (5.02) kg/m^2 ^and mean (SD) BMI-for-age z-score was -0.3 (1.5). Overall, 17% (n = 316) children were overweight and 7.5% (n = 140) were obese.

### Association of dietary behaviors, physical activity and sedentary lifestyle with overweight and obesity

**Figure 1:**
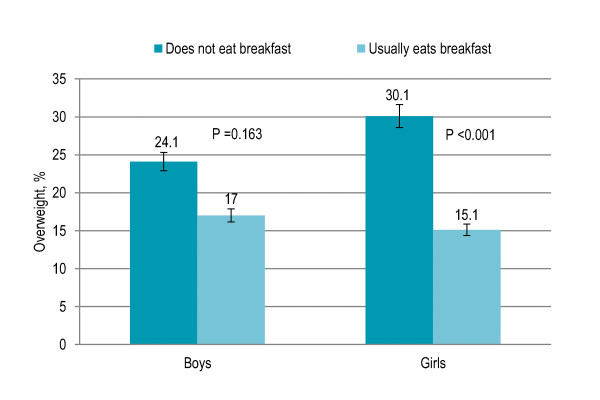
**Gender-specific trend in breakfast eating pattern and prevalence of overweight among Pakistani primary school boys (n = 977) and girls (n = 883)**.

**Figure 2:**
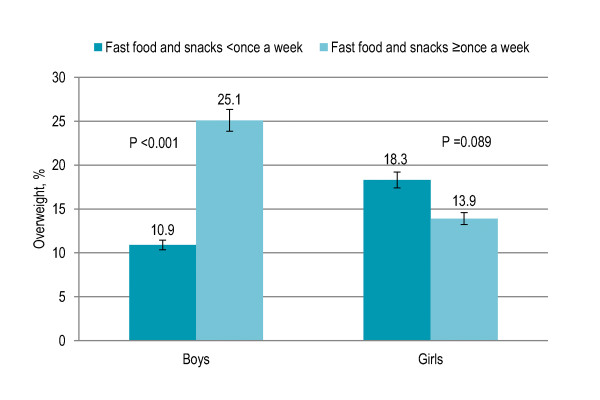
**Gender-specific trend in fast food and snacks eating pattern and prevalence of overweight among Pakistani primary school boys (n = 977) and girls (n = 883)**.

Eight percent children did not have breakfast and were significantly more likely to be overweight (15% vs. 9%) and obese (13% vs. 7%) than those having breakfast (P = 0.002). Skipping breakfast was associated with overweight among girls (P < 0.001) but the association was not significant among boys (P = 0.163) [Figure 1]. Seventy-nine percent children had lunch at school while others (21%) had lunch at home after school and no significant association was observed with overweight and obesity. Sixty-five percent children had supper with family more than five times a week while the rest had it four to five times (17%), one to three times (10%) or less than once a week (8%) and there was no significant association with overweight and obesity. Fifty-seven percent children had fast food and snacks less than once a week while others had one to two times (30%) or more than three times a week (13%). Children eating fast food and snacks were significantly more likely to be overweight and obese (P = 0.027). Eating fast food and snacks once or more a week was associated with overweight among boys (P < 0.001) but the association was not significant among girls (0.089) [Figure 2]. [Table 1]

**Table 1:** Association of dietary behaviors with overweight and obesity among Pakistani primary school children

	Total Sample (n = 1860)	Normal (n = 1544)	Overweight (n = 176)	Obese (n = 140)		
			
Characteristics	n (%)	n (%)	n (%)	n (%)	**χ**^**2**^	P value
**Breakfast**						
Usually eat	1719 (92.4)	1442 (83.9)	155 (9.0)	122 (7.1)	12.41	0.002
Does not eat	141 (7.6)	102 (72.3)	21 (14.9)	18 (12.8)		
**Lunch**						
Eat at school	1468 (78.9)	1216 (82.8)	138 (9.4)	114 (7.8)	0.58	0.747
Eat at home	392 (21.1)	328 (83.7)	38 (9.7)	26 (6.6)		
**Family Supper**						
< once/week	151 (8.1)	130 (86.1)	8 (5.3)	13 (8.6)	9.86	0.131
1-3 times/week	179 (9.6)	154 (86.0)	15 (8.4)	10 (5.6)		
4-5 times/week	316 (17.0)	263 (83.2)	37 (11.7)	16 (5.1)		
> 5 times/week	1214 (65.3)	997 (82.1)	116 (9.6)	101 (8.3)		
**Fast food and snacks**						
< once/week	1062 (57.1)	907 (85.4)	83 (7.8)	72 (6.8)	10.98	0.027
1-2 times/week	549 (29.5)	441 (80.3)	62 (11.3)	46 (8.4)		
≥ 3 times/week	249 (13.4)	196 (78.7)	31 (12.4)	22 (8.8)		

**Figure 3:**
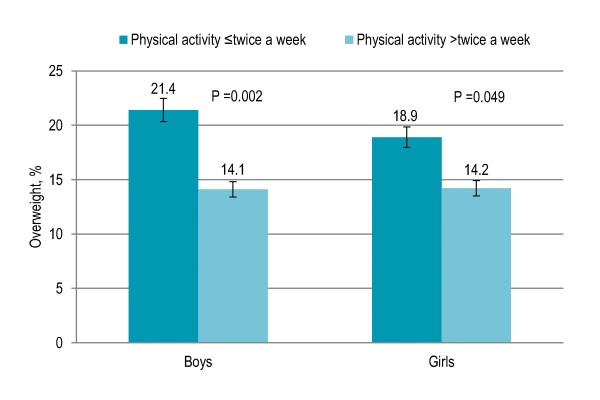
**Gender-specific trend in physical activity and prevalence of overweight among Pakistani primary school boys (n = 977) and girls (n = 883)**. Physical activity included running or jogging, cycling, housework or yard work, and sports involving physical movement (see details in methods).

**Figure 4:**
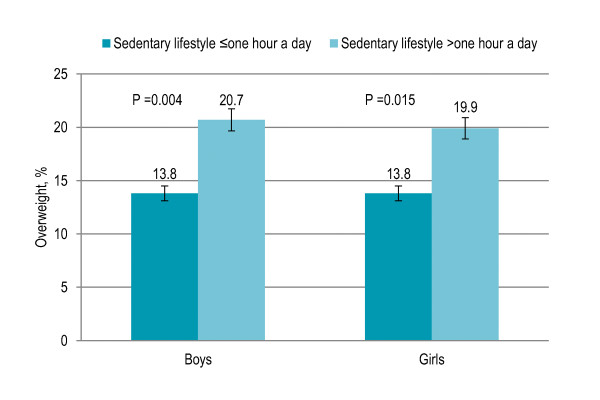
**Gender-specific trend in sedentary lifestyle and prevalence of overweight among Pakistani primary school boys (n = 977) and girls (n = 883)**. Sedentary lifestyle included television viewing, working on computer and playing video games.

Forty-seven percent children participated in physical activity twice or less a week while others participated three to four times (20%) or five to seven times a week (33%). Physical activity more than twice a week showed a significant inverse association with overweight and obesity (P = 0.001). Among both boys and girls, physical activity more than twice a week was associated with overweight (boys P = 0.002, girls P = 0.049) [Figure 3]. Thirty-two percent children, of whom 92.5% were rural and urban with low SES, traveling to and from school by walk or bike were significantly less likely to be overweight and obese (P < 0.001) than the rest (68%) who were driven to and from school. Fifty-one percent children were involved in sedentary lifestyle one hour or less a day while others were involved more than one hour to three hours (39%) or more than three hours to six hours a day (10%). The risk of being overweight and obese significantly increased with the involvement in sedentary lifestyle (P = 0.003). Among both boys and girls, sedentary lifestyle more than one hour a day was associated with overweight (boys P = 0.004, girls P = 0.015) [Figure 4]. [Table 2]

**Table 2:** Association of physical activity and sedentary lifestyle with overweight and obesity among Pakistani primary school children

	Total Sample (n = 1860)	Normal (n = 1545)	Overweight (n = 176)	Obese (n = 140)		
			
Characteristics	n (%)	n (%)	n (%)	n (%)	**χ**^**2**^	P value
**Physical activity**^**a**^					19.52	0.001
≤ 2 times/week	872 (46.9)	696 (79.8)	89 (10.2)	87 (10.0)		
> 2 times - 4 times/week	365 (19.6)	323 (88.5)	29 (7.9)	13 (3.6)		
> 4 times - 7 times/week	623 (33.5)	525 (84.3)	58 (9.3)	40 (6.4)		
**Sedentary lifestyle**^**b**^					15.83	0.003
≤ 1 hour/day	951 (51.1)	820 (86.2)	76 (8.0)	55 (5.8)		
> 1 hour - 3 hours/day	730 (39.2)	585 (80.1)	80 (11.0)	65 (8.9)		
> 3 hours - 6 hours/day	179 (9.6)	139 (77.7)	20 (11.2)	20 (11.2)		

^a^Physical activity included running or jogging, cycling, housework or yard work, and sports involving physical movement (see details in methods)

^b^Sedentary lifestyle included television viewing, working on computer and playing video games

In linear regression analysis, skipping breakfast (P < 0.001), eating fast food and snacks (P = 0.001) and sedentary lifestyle (P < 0.001) showed a significant independent positive association with BMI while physical activity showed a significant independent inverse association (P = 0.001) [Table 3]. In multivariate logistic regression analysis, skipping breakfast (aOR 1.82, 95% CI 1.22-2.71), eating fast food and snacks once or more a week (OR 1.41, 95% CI 1.07-1.86), physical activity more than twice a week (aOR 0.49, 95% CI 0.34-0.70) and sedentary lifestyle more than one hour a day (aOR 1.56, 95% CI 1.19-2.03) were significant independent predictors of being overweight [Table 4]. Multivariate logistic regression analysis, controlled for age and gender, was conducted to explore the associations among dietary behaviors, physical activity and sedentary lifestyle. Skipping breakfast had a significant independent inverse association with physical activity more than twice a week (aOR 0.63, 95% CI 0.45-0.89) and eating fast food and snacks once or more a week had a significant independent positive association with sedentary lifestyle more than one hour a day (aOR 1.79, 95% CI 1.49-2.16) [Table 5].

**Table 3:** Linear regression analysis of dietary behaviors, physical activity and sedentary lifestyle with BMI among Pakistani primary school children (n = 1860)^a,^^b^

Characteristics	Regression coefficient (95% CI)	Standard error
Skipping breakfast	1.87 (1.12 to 2.62)^† ^	0.38
Eating fast food and snacks	0.48 (0.20 to 0.76)^‡^	0.14
Physical activity^c^	-0.38 (-0.61 to -0.16)^‡^	0.12
Sedentary lifestyle^d^	0.63 (0.32 to 0.93)^† ^	0.16

^† ^P < 0.001, ^‡^P = 0.001

^a^The model is controlled for age and gender

^b^R^2 ^= 0.253

^c^Physical activity included running or jogging, cycling, housework or yard work, and sports involving physical movement (see details in methods)

^d^Sedentary lifestyle included television viewing, working on computer and playing video games

**Table 4:** Logistic regression analysis of dietary behaviors, physical activity and sedentary lifestyle associated with overweight among Pakistani primary school children (n = 1860)

	Overweight, including obese (n = 316)	
	
Characteristics	Crude OR (95% CI)	**Adjusted OR (95% CI)**^**a**^
**Diet-related factors**		
**Breakfast**		
Usually eat	Ref.	Ref.
Does not eat	2.00 (1.35-2.94)^‡^	1.82 (1.22-2.71)^‡^
**Fast food and snacks**		
< once/week	Ref.	Ref.
1-2 times/week	1.43 (1.09-1.88)^‡^	1.41 (1.07-1.86)*
≥ 3 times/week	1.58 (1.12-2.24)*	1.56 (1.09-2.24)*
**Activity-related factors**		
**Physical activity**^**b**^		
≤ 2 times/week	Ref.	Ref.
> 2 times - 4 times/week	0.51 (0.36-0.74)^† ^	0.49 (0.34-0.70)^† ^
> 4 times - 7 times/week	0.74 (0.56-0.97)*	0.71 (0.54-0.94)*
**Sedentary lifestyle**^**c**^		
≤ 1 hour/day	Ref.	Ref.
> 1 hour - 3 hours/day	1.55 (1.20-2.01)^‡^	1.56 (1.19-2.03)^‡^
> 3 hours - 6 hours/day	1.80 (1.21-2.68)^‡^	1.69 (1.12-2.56)*

^† ^P < 0.001, ^‡^P < 0.01, *P < 0.05

^a^The model is controlled for age and gender

^b^Physical activity included running or jogging, cycling, housework or yard work, and sports involving physical movement (see details in methods)

^c^Sedentary lifestyle included television viewing, working on computer and playing video games

**Table 5:** Logistic regression analysis for association between dietary behaviors, physical activity and sedentary lifestyle among Pakistani primary school children (n = 1860)

	**Physical activity > twice a week (n = 988)**^**a**^		**Sedentary lifestyle > one hour a day (n = 909)**^**b**^	
	
Characteristics	Crude OR (95% CI)	**Adjusted OR (95% CI)**^**c**^	Crude OR (95% CI)	**Adjusted OR (95% CI)**^**c**^
**Skipping breakfast (n = 141)**	0.65 (0.46-0.92)*	0.63 (0.45-0.89)^‡^	1.32 (0.94-1.89)	1.35 (0.95-1.92)
**Eating fast food and snacks **≥ **once a week (n = 798)**	1.12 (0.93-1.35)	1.07 (0.88-1.29)	1.80 (1.50-2.18)^† ^	1.79 (1.49-2.16)^† ^

^† ^P < 0.001, ^‡^P < 0.01, *P < 0.05

^a^Physical activity included running or jogging, cycling, housework or yard work, and sports involving physical movement (see details in methods)

^b^Sedentary lifestyle included television viewing, working on computer and playing video games

^c^The model is controlled for age and gender

### Socio-demographic correlates of dietary behaviors, physical activity and sedentary lifestyle independently associated with higher BMI and overweight

Skipping breakfast, eating fast food and snacks once or more a week, physical activity more than twice a week and sedentary lifestyle more than one hour a day were independently associated with overweight and higher BMI. Female gender (P = 0.005), urban area with high SES (P < 0.001), high-income neighborhoods (P < 0.001), higher parental education (P < 0.001) and fewer siblings (P = 0.001) were significantly associated with skipping breakfast [Table 6], and female gender remained the only significant independent predictor in multivariate logistic regression analysis (aOR 1.50, 95% CI 1.04-2.16) [Table 7]. Significant correlates of eating fast food and snacks once or more a week were male gender (P = 0.017), urban area with high SES (P < 0.001), high-income neighborhoods (P < 0.001), higher parental education (P < 0.001), both parents working (P < 0.001), fewer siblings (P = 0.001) and less crowded housing (P < 0.001) [Table 6]. In multivariate logistic regression analysis, male gender (aOR 1.64, 95% CI 1.33-2.02), urban area with high SES (aOR 5.09, 95% CI 3.02-8.60) and higher parental education (aOR 1.74, 95% CI 1.12-2.68) were significant independent predictors of eating fast food and snacks once or more a week [Table 8]. The rural area with low/disadvantaged SES (P < 0.001) and low-income neighborhoods (P = 0.017) were significantly associated with physical activity more than twice a week [Table 9], and the rural area with low/disadvantaged SES (aOR 2.51, 95% CI 1.71-3.68) remained the only significant independent predictor in multivariate logistic regression analysis [Table 10]. Significant correlates of sedentary lifestyle more than one hour a day were male gender (P = 0.001), urban area with high SES (P < 0.001), high-income neighborhoods (P < 0.001), higher parental education (P < 0.001), fewer siblings (P = 0.001) and less crowded housing (P < 0.001) [Table 9]. In multivariate logistic regression analysis, male gender (aOR 1.60, 95% CI 1.31-1.95), urban area with low SES (aOR 1.46, 95% CI 1.02-2.09), high-income neighborhoods (aOR 1.52, 95% CI 1.02-2.25), higher parental education (aOR 1.55, 95% CI 1.03-2.34) and fewer siblings (aOR 1.38, 95% CI 1.10-1.73) were significant independent predictors of sedentary lifestyle more than one hour a day [Table 11].

**Table 6:** Socio-demographic correlates of dietary behaviors independently associated with higher BMI and overweight among Pakistani primary school children

	Total Sample (n = 1860)	Skipping breakfast (n = 141)			**Eating fast food and snacks **≥ **once a week (n = 798)**		
	
Characteristics	n (%)	n (%)	**χ**^**2**^	P value	n (%)	**χ**^**2**^	P value
**Gender**							
Boys	977 (52.5)	58 (5.9)	7.94	0.005	446 (45.6)	6.34	0.012
Girls	883 (47.5)	83 (9.4)			352 (39.9)		
**Area and socioeconomic status (SES)**							
Urban, high SES	465 (25.0)	68 (14.6)	50.79	< 0.001	274 (58.9)	233.88	< 0.001
Urban, middle SES	465 (25.0)	26 (5.6)			254 (54.6)		
Urban, low SES	465 (25.0)	34 (7.3)			205 (44.1)		
Rural, low/disadvantaged SES	465 (25.0)	13 (2.8)			65 (14.0)		
**Neighborhood income**							
Low	651 (35.0)	22 (3.4)	33.72	< 0.001	154 (23.7)	151.87	< 0.001
Middle	910 (48.9)	78 (8.6)			480 (52.7)		
High	299 (16.1)	41 (13.7)			164 (54.8)		
**Parental education**							
Illiterate	366 (19.7)	13 (3.6)	23.78	< 0.001	64 (17.5)	176.82	< 0.001
High school	496 (26.7)	25 (5.0)			177 (35.7)		
College	531 (28.5)	54 (10.2)			307 (57.8)		
Higher education	467 (25.1)	49 (10.5)			250 (53.5)		
**Parental working status**							
Father only	1465 (78.8)	111 (7.6)	0.00	0.990	590 (40.3)	19.48	< 0.001
Both parents	395 (21.2)	30 (7.6)			208 (52.7)		
**Number of siblings**							
No	26 (1.4)	2 (7.7)	14.67	0.001	13 (50.0)	69.47	< 0.001
1-3	1008 (54.2)	98 (9.7)			519 (51.5)		
> 3	826 (44.4)	41 (5.0)			266 (32.2)		
**Number of persons in child's living room**							
No	116 (6.2)	10 (8.6)	1.62	0.444	70 (60.3)	51.45	< 0.001
1-3	791 (42.5)	66 (8.3)			392 (49.6)		
> 3	953 (51.2)	65 (6.8)			336 (35.3)		

**Table 7:** Logistic regression analysis of socio-demographic factors associated with skipping breakfast among Pakistani primary school children (n = 1860)

	Skipping breakfast (n = 141)	
	
Characteristics	Crude OR (95% CI)	**Adjusted OR (95% CI)**^**a**^
**Gender**		
Boys	Ref.	Ref.
Girls	1.64 (1.16-2.33)^‡^	1.50 (1.04-2.16)*
**Area and socioeconomic status (SES)**		
Urban, high SES	5.96 (3.24-10.94)^† ^	2.34 (0.86-6.36)
Urban, middle SES	2.06 (1.05-4.06)*	1.02 (0.37-2.79)
Urban, low SES	2.74 (1.43-5.27)^‡^	1.68 (0.70-4.01)
Rural, low/disadvantaged SES	Ref.	Ref.
**Neighborhood income**		
Low	Ref.	Ref.
Middle	2.68 (1.65-4.35)^† ^	1.64 (0.88-3.05)
High	4.54 (2.65-7.78)^† ^	1.94 (0.93-4.07)
**Parental education**		
Illiterate	Ref.	Ref.
High school	1.44 (0.73-2.86)	0.95 (0.43-2.13)
College	3.07 (1.65-5.72)^† ^	1.33 (0.56-3.15)
Higher education	3.18 (1.70-5.96)^† ^	1.27 (0.52-3.08)
**Number of siblings**		
No	1.60 (0.37-6.98)	0.85 (1.89-3.85)
1-3	2.06 (1.42-3.01)^† ^	1.33 (0.87-2.03)
> 3	Ref.	Ref.

^† ^P < 0.001, ^‡^P < 0.01, *P < 0.05

^a^The model is controlled for age

**Table 8:** Logistic regression analysis of socio-demographic factors associated with eating fast food and snacks **≥ **once a week among Pakistani primary school children (n = 1860)

	**Eating fast food and snacks **≥ **once a week (n = 798)**	
	
Characteristics	Crude OR (95% CI)	**Adjusted OR (95% CI)**^**a**^
**Gender**		
Boys	1.27 (1.05-1.52)*	1.64 (1.33-2.02)^† ^
Girls	Ref.	Ref.
**Area and socioeconomic status (SES)**		
Urban, high SES	8.83 (6.41-12.17)^† ^	5.09 (3.02-8.60)^† ^
Urban, middle SES	7.41 (5.38-10.20)^† ^	3.88 (2.36-6.37)^† ^
Urban, low SES	4.85 (3.52-6.68)^† ^	3.86 (2.54-5.87)^† ^
Rural, low/disadvantaged SES	Ref.	Ref.
**Neighborhood income**		
Low	Ref.	Ref.
Middle	3.60 (2.88-4.50)^† ^	1.35 (1.00-1.83)
High	3.92 (2.93-5.24)^† ^	1.23 (0.81-1.87)
**Parental education**		
Illiterate	Ref.	Ref.
High school	2.62 (1.89-3.63)^† ^	1.11 (0.75-1.64)
College	6.47 (4.70-8.91)^† ^	1.74 (1.12-2.68)*
Higher education	5.44 (3.93-7.53)^† ^	1.38 (0.87-2.17)
**Parental working status**		
Father only	Ref.	Ref.
Both parents	1.65 (1.32-2.06)^† ^	1.26 (0.98-1.61)
**Number of siblings**		
No	2.11 (0.96-4.60)	0.68 (0.30-1.54)
1-3	2.23 (1.85-2.71)^† ^	0.97 (0.77-1.22)
> 3	Ref.	Ref.
**Number of persons in child's living room**		
No	2.79 (1.88-4.15)^† ^	1.36 (0.88-2.09)
1-3	1.80 (1.49-2.19)^† ^	1.15 (0.92-1.43)
> 3	Ref.	Ref.

^† ^P < 0.001, *P < 0.05

^a^The model is controlled for age

**Table 9:** Socio-demographic correlates of physical activity and sedentary lifestyle independently associated with higher BMI and overweight among Pakistani primary school children

	Total Sample (n = 1860)	**Physical activity > twice a week (n = 988)**^**a**^			**Sedentary lifestyle > one hour a day (n = 909)**^**b**^		
	
Characteristics	n (%)	n (%)	**χ**^**2**^	P value	n (%)	**χ**^**2**^	P value
**Gender**							
Boys	977 (52.5)	538 (55.1)	3.14	0.077	512 (52.4)	10.29	0.001
Girls	883 (47.5)	450 (51.0)			397 (45.0)		
**Area and socioeconomic status (SES)**							
Urban, high SES	465 (25.0)	224 (48.2)	36.39	< 0.001	280 (60.2)	60.32	< 0.001
Urban, middle SES	465 (25.0)	229 (49.2)			229 (49.2)		
Urban, low SES	465 (25.0)	232 (49.9)			237 (51.0)		
Rural, low/disadvantaged SES	465 (25.0)	303 (65.2)			163 (35.1)		
**Neighborhood income**							
Low	651 (35.0)	374 (57.5)	8.16	0.017	248 (38.1)	50.83	< 0.001
Middle	910 (48.9)	468 (51.4)			482 (53.0)		
High	299 (16.1)	146 (48.8)			179 (59.9)		
**Parental education**							
Illiterate	366 (19.7)	212 (57.9)	7.30	0.063	133 (36.3)	62.12	< 0.001
High school	496 (26.7)	273 (55.0)			205 (41.3)		
College	531 (28.5)	264 (49.7)			305 (57.4)		
Higher education	467 (25.1)	239 (51.2)			266 (57.0)		
**Parental working status**							
Father only	1465 (78.8)	785 (53.6)	0.60	0.439	732 (50.0)	3.31	0.069
Both parents	395 (21.2)	203 (51.4)			177 (44.8)		
**Number of siblings**							
No	26 (1.4)	15 (57.7)	0.23	0.893	17 (65.4)	46.99	< 0.001
1-3	1008 (54.2)	534 (53.0)			561 (55.7)		
> 3	826 (44.4)	439 (53.1)			331 (40.1)		
**Number of persons in child's living room**							
No	116 (6.2)	51 (44.0)	4.24	0.120	73 (62.9)	16.96	< 0.001
1-3	791 (42.5)	422 (53.4)			407 (51.5)		
> 3	953 (51.2)	515 (54.0)			429 (45.0)		

^a^Physical activity included running or jogging, cycling, housework or yard work, and sports involving physical movement (see details in methods)

^b^Sedentary lifestyle included television viewing, working on computer and playing video games

**Table 10:** Logistic regression analysis of socio-demographic factors associated with physical activity > twice a week among Pakistani primary school children (n = 1860)

	**Physical activity > twice a week (n = 988)**^**a**^	
	
Characteristics	Crude OR (95% CI)	**Adjusted OR (95% CI)**^**b**^
**Area and socioeconomic status (SES)**		
Urban, high SES	Ref.	Ref.
Urban, middle SES	1.04 (0.81-1.35)	1.01 (0.75-1.35)
Urban, low SES	1.07 (0.83-1.39)	1.11 (0.81-1.53)
Rural, low/disadvantaged SES	2.01 (1.55-2.62)^† ^	2.51 (1.71-3.68)^† ^
**Neighborhood income**		
Low	1.42 (1.08-1.86)*	0.81 (0.55-1.19)
Middle	1.11 (0.85-1.44)	1.09 (0.80-1.47)
High	Ref.	Ref.

^† ^P < 0.001, *P < 0.05

^a^Physical activity included running or jogging, cycling, housework or yard work, and sports involving physical movement (see details in methods)

^b^The model is controlled for age and gender

**Table 11:** Logistic regression analysis of socio-demographic factors associated with sedentary lifestyle > one hour a day among Pakistani primary school children (n = 1860)

	**Sedentary lifestyle > one hour a day (n = 909)**^**a**^	
	
Characteristics	Crude OR (95% CI)	**Adjusted OR (95% CI)**^**b**^
**Gender**		
Boys	1.35 (1.12-1.62)^‡^	1.60 (1.31-1.95)^† ^
Girls	Ref.	Ref.
**Area and socioeconomic status (SES)**		
Urban, high SES	2.80 (2.15-3.66)^† ^	1.16 (0.72-1.86)
Urban, middle SES	1.80 (1.38-2.34)^† ^	0.82 (0.53-1.28)
Urban, low SES	1.93 (1.48-2.51)^† ^	1.46 (1.02-2.09)*
Rural, low/disadvantaged SES	Ref.	Ref.
**Neighborhood income**		
Low	Ref.	Ref.
Middle	1.83 (1.49-2.25)^† ^	1.36 (1.02-1.82)*
High	2.42 (1.83-3.21)^† ^	1.52 (1.02-2.25)*
**Parental education**		
Illiterate	Ref.	Ref.
High school	1.23 (0.94-1.63)	0.94 (0.68-1.29)
College	2.36 (1.80-3.11)^† ^	1.61 (1.09-2.38)*
Higher education	2.32 (1.75-3.07)^† ^	1.55 (1.03-2.34)*
**Number of siblings**		
No	2.83 (1.24-6.41)*	1.55 (0.66-3.65)
1-3	1.88 (1.56-2.62)^† ^	1.38 (1.10-1.73)^‡^
> 3	Ref.	Ref.
**Number of persons in child's living room**		
No	2.07 (1.39-3.09)^† ^	1.39 (0.90-2.14)
1-3	1.30 (1.07-1.56)^‡^	0.98 (0.79-1.21)
> 3	Ref.	Ref.

^† ^P < 0.001, ^‡^P < 0.01, *P < 0.05

^a^Sedentary lifestyle included television viewing, working on computer and playing video games

^b^The model is controlled for age

## Discussion

Dietary behaviors, physical activity and sedentary lifestyle associated with the risk of being overweight and obese and having higher BMI among Pakistani primary school children, and their socio-demographic correlates, were described in the present study. Children who skipped breakfast were more likely to be overweight and obese as compared to those who regularly had their breakfast. Gender-specific trend in breakfast skipping and prevalence of overweight revealed that the relation was significant among girls but not boys. After adjusting for age, gender and all factors, skipping breakfast was a significant independent predictor of higher BMI and overweight. Previous studies have reported lower risk of overweight and lower BMI among children having breakfast regularly compared with those who frequently skip breakfast [20-24]. Female gender and living in urban area were associated with skipping breakfast, consistent with previous studies [24,25]. Higher social class, represented by socio-demographic characteristics of the child including urban area with high SES, high-income neighborhoods, higher parental education and fewer siblings, was associated with breakfast skipping behavior. Female gender was independent predictor of skipping breakfast. The reason for breakfast skipping among females might be a higher concern for body image [26]. Skipping breakfast had significant independent inverse association with physical activity, further contributing to increased adiposity, consistent with previous literature [27,28]. A school-based breakfast program, especially in the urban area and among girls, could be beneficial to improve the nutritional status. Media-based health education campaigns tailored to local circumstances and targeting the higher social class could be initiated.

Children who had lunch at school and did not eat supper together with their family have been reported at an increased risk of being overweight [29,30]; however, no significant association was observed in the present study. Higher frequency of eating fast food and snacks was associated with overweight and obesity, consistent with previous literature [30-35]. Gender-specific trend in eating fast food and snacks and prevalence of overweight revealed that the relation was significant among boys but not girls. Eating fast food and snacks showed a significant independent association with higher BMI and risk of being overweight after adjusting for age, gender and all factors. Higher consumption of fast food and snacks was observed among boys, urban children and those having higher social class depicted by high SES, high-income neighborhoods, higher parental education, both parents working, fewer siblings and less crowded housing, in line with previous studies [33-36]. Male gender, urban area with high SES and higher parental education were significant independent predictors of eating fast food and snacks. A possible explanation for the gender disparity may be higher nutrition knowledge among girls owing to their high concern with self-perception of body image [26], and the socio-cultural matrix in South Asia that prioritize boys in feeding practices. Parents are less likely to encourage sons to lose weight perhaps because of the larger, more muscular ideal male body shape [37]. Eating fast food and snacks had a significant independent association with sedentary lifestyle, consistent with previous literature [38]. Restriction on television advertising of fast foods to children, applying taxes on energy-dense foods and food labeling have proved to be useful in the developed countries [39].

Physical activity more than twice a week had a significant independent inverse relationship with overweight and BMI. It is well established that physical activity increases a child's energy expenditure and leads to a lower risk of overweight [2,30,39-43]. Correlates of higher physical activity were the rural area with low/disadvantaged SES and low-income neighborhoods. Living in the rural area was significant independent predictor of higher physical activity. Children who travelled actively to and from school by walk or bike were significantly less likely to be overweight and obese than those who were driven to and from school, consistent with previous findings [2,44,45]. Built environment, walking and cycling paths, public open spaces and parks, safe and inexpensive recreation and sports centers and an improved quality and duration of physical education in schools can be considered for increasing physical activity [2,39].

Sedentary lifestyle that included television viewing, working on computer and playing video games showed a significant independent association with higher BMI and risk of being overweight, consistent with previous studies [38,40-43,46,47]. Increased television viewing has been associated with a higher energy intake [38]. Sedentary lifestyle was more common among boys, urban children and those having higher social class depicted by high SES, high-income neighborhoods, higher parental education, fewer siblings and less crowded housing. Sedentary lifestyle has been previously associated with the urban area and high SES [47]. Behavioral interventions have shown promising results to reduce the time spent in sedentary lifestyle, especially television viewing, among children [48]. Physical activity and sedentary lifestyle were independently associated with overweight, consistent with a recent study, and preventive strategies may need to target sedentary lifestyle and physical activity separately [49].

These findings can be generalized to South Asian primary school children sharing the same genetic and environmental factors with the study sample. Cross-sectional nature of the study should be considered when interpreting the findings. Future longitudinal studies involving these factors are warranted to establish the temporal nature and causality of these associations among both boys and girls. Although data collection followed a standard protocol, digital scales were not used. Variability in the data ascertainment may have introduced error into the prevalence estimates; however, we do not anticipate large or systematic differences. The responses were based on self-recall and recall bias is a recurring problem with any self-report survey; however, this limitation may be tolerable considering the goals of the questionnaire and previous studies support the validity of this methodology in school-aged children [12,13,15,16].

## Conclusions

Dietary behaviors, physical activity and sedentary lifestyle are independent predictors of overweight and higher BMI among Pakistani primary school children, and are significantly affected by the child's socio-demographic characteristics. In both the developed and developing countries, childhood obesity epidemic has been attributed to a growing obesogenic environment that essentially facilitates the intake of energy-dense foods while restricting or inhibiting all activities demanding high energy expenditures [50]. Programs designed to encourage healthy eating behaviors, increase physical activity and reduce sedentary lifestyle have been shown to decrease childhood obesity [30,48,51]. Family-based and culturally relevant behavioral interventions that have been effective in weight management need to be tested and implemented in the resource-poor developing country settings [5,48,52]. A National strategy for diet and physical activity should be developed and a preventive program should be initiated, considering the dietary behaviors, physical activity and sedentary lifestyle associated with childhood obesity and the socio-demographic factors affecting these.

## Competing interests

The authors declare that they have no competing interests.

## Authors' contributions

MUM, principal investigator, conceived and implemented the study, analyzed and interpreted the data, prepared the manuscript and supervised the entire project. SG and KM contributed to the study analysis, interpretation and manuscript preparation. US contributed to the study conception, implementation and analysis. MAS and JA oversaw the study conception, implementation and manuscript preparation. All authors read and approved the final manuscript.
